# Evaluation of enamel surface integrity after orthodontic bracket debonding: comparison of three different system

**DOI:** 10.1186/s12903-024-04138-4

**Published:** 2024-03-20

**Authors:** Labib Ghaleb, Naseem Ali Al-Worafi, Ahmed Thawaba, Abbas Ahmed Abdulqader, Aqlan Alkamel, Yaser Abdo, Zhao Yang, Nashwan Noman, Maged Ali Al-Aroomi, Tian Yulou

**Affiliations:** 1 Department of Orthodontics, School and Hospital of Stomatology, China Medical University, Shenyang, 110002 China; 2 Department of orthodontics, College of dentistry, Manila central university, Manila, Philippines; 3 Orthodontic Department, Faculty of Dentistry, Mansoura University, Mansoura, Egypt; 4 Department of Orthodontics and Dentofacial Orthopedics, College of Dentistry, West China Hospital of Stomatology, Sichuan University, Chengdu, China; 5 Department of Oral and Maxillofacial Surgery, Faculty of Dentistry, Taiz University, Taiz, Yemen; 6 Department of Oral and Maxillofacial Surgery, Faculty of Dentistry, Ibb University, Ibb, Yemen

**Keywords:** Surface roughness, Debonding, Brackets, Orthodontic adhesive, Magnifying loupe

## Abstract

**Objective:**

This study aimed to evaluate enamel surface integrity and time consumed during residual cement removal after bracket debonding using different adhesive removal burs with and without a dental loupe.

**Material and Methods:**

Sixty human-extracted premolars were collected, cleaned, mounted, and prepared for orthodontic bracket bonding. Teeth were randomly divided into three main groups (*n* = 20) based on the adhesive removal method: tungsten carbide system (TC), sof-lex discs system (SD), and diamond system (DB) groups. Then, each group was subdivided into two subgroups (naked eye and magnifying loupe subgroups). The brackets were bonded and then debonded after 24 h, and the Adhesive Remnant Index (ARI) was assessed. The adhesive remnants were removed by different systems, and the final polishing was performed by Silicone OneGloss. The enamel surface roughness was evaluated before bracketing (T0), after residual cement removal (T1), and finally after polishing (T2) using surface Mitutoyo SJ-210 profilometry and Scanning Electron Microscopy (SEM) to determine the Enamel Damage Index (EDI) score. The time consumed for adhesive removal was recorded in seconds.

**Results:**

The Kruskal Wallis test showed a statistically significant difference in roughness values at T1 compared to T2 between subgroups (*p* < 0.001). When comparing EDI at T1 and T2, the Kruskal–Wallis H-test showed statistically significant differences in all subgroups. The pairwise comparisons revealed that EDI scores showed a statistically significant difference at T1 and T2 between DB vs. TC and SD (*p* = 0.015) but not between TC vs. SD (*p* = 1.000), indicating the highest roughness value observed in the DB group. The time for cement removal was significantly shorter in the magnifying loupe group than in the naked eye group and was shortest with the TC group, whereas the time was the longest with the DB group (*p* < 0.05).

**Conclusion:**

All three systems were clinically satisfactory for residual orthodontic adhesive removal. However, TC system produced the lowest enamel roughness, while the DB system created the greatest. The polishing step created smoother surfaces regardless of the systems used for resin removal.

**Supplementary Information:**

The online version contains supplementary material available at 10.1186/s12903-024-04138-4.

## Introduction

Dental enamel, the most mineralized tissue in the human body, forms the external protective layer of a tooth’s anatomical crown [1]. Fixed orthodontic brackets are temporary appliances attached to the teeth for a certain period, depending on the severity of the malocclusion, and need to be removed at the end of treatment [2]. Significant efforts are made to minimize the risk of enamel surface damage and restore the enamel surface promptly after orthodontic debonding and resin removal [3]. If the roughened areas are left untreated, they may promote dental plaque accumulation, subsequent enamel demineralization, and decay. Another concern is the discoloration of composite remnants over time, causing an unaesthetic appearance [4]. Therefore, the primary goal of bracket debonding is to remove orthodontic debonding and adhesive remnants from tooth surfaces without causing iatrogenic damage.

Many methods were used for bracket debonding, such as manual, rotary instruments, ultrasonic, air abrasion by sandblast, and lasers [5–7]. Different factors, including the bur type, its rotational speed, the number of blades, and the material composition, influence the extent of the enamel damage during adhesive removal [8, 9]. Tungsten Carbide system (TC) is perfect for cutting ductile materials such as composite resins. The rotation of these burs generates high shear forces between the bur’s blades and the resin surface, resulting in the plastic plowing of the resin. Many TC are available on the market, and almost all of them have been recommended for adhesive removal in the literature [10]. Employ the SD system, ranging from coarse to extra fine, to attain a smooth enamel surface. These discs can be used alone or in conjunction with TC system. While it is feasible to use Sof-lex discs independently for adhesive removal and enamel polishing, this method is more time-consuming than when combined with burs. Nonetheless, SD system is effective in easily flattening the enamel surface [11].

Dental loupes are extensively employed in dentistry due to their significant benefits in enhancing ergonomics. This widely adopted tool not only aids in magnification but also contributes to better posture and reduced strain for dental professionals during procedures [12, 13]. Orthodontic treatments require a clear view of one or both dental arches, and using magnifying loupes might help with certain tasks like placing brackets or removing adhesive after debonding by giving better visual control [14]. Based on our knowledge, no technique has been shown to thoroughly and efficiently remove residual adhesives without causing at least some minor damage to the enamel. The null hypothesis is that there are no differences in the integrity of the enamel surface after orthodontic deboning adhesive removal after using three different burs with and without the aid of a dental loupe. It also suggests no significant difference in the time taken for adhesive removal. Therefore, this study worked to prove or reject this null hypothesis.

## Materials and methods

The study protocol was approved by the ethical and research committee at China Medical University, School of Stomatology, and has been conducted in full accordance with the Declaration of Helsinki. The sample size was calculated using G* Power software (v3.1.3; Franz Faul, Universität Kiel, Germany) which depends on the effect size = 0.80, an alpha value of 0.05, and a power of 95%. The result showed that at least 15 samples were required in each group based on the study conducted by Thawaba et al. [15]. However, the sample size was raised to 20 samples for each study group.

### Sample selection and preparation

**Fig. 1:**
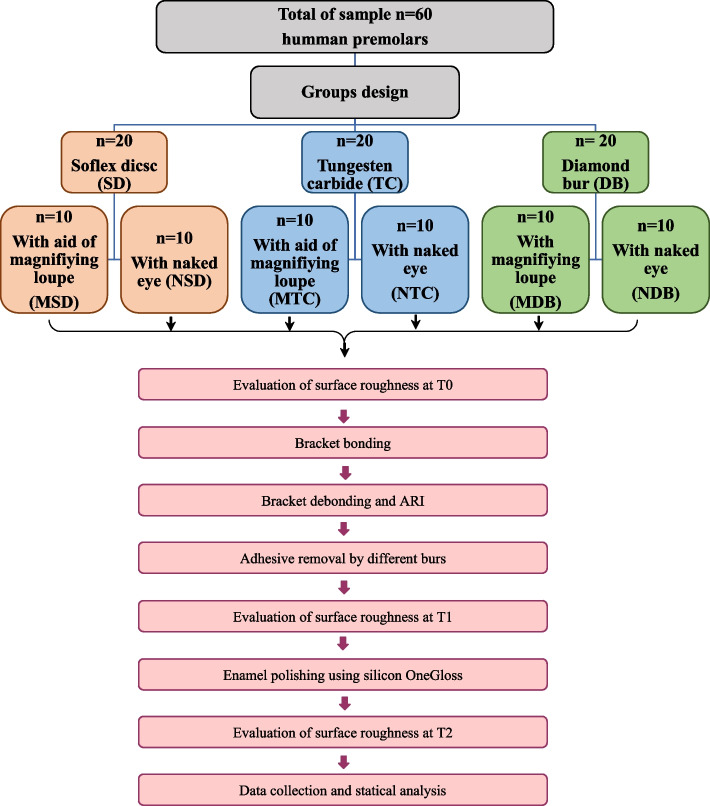
A diagram that illustrates the experimental design and how the group was assigned

This in-vitro study was conducted on 60 freshly extracted premolar teeth collected from the outpatient clinics. The teeth were carefully inspected using light to ensure they are healthy buccal surfaces without any visible damage like chips, cracks, or previous dental work (e.g., no braces have been attached to them before). To simplify the process of bonding procedures, the root part of each tooth was fixed into blocks made of acrylic material. The total sample was randomly divided into three equal groups (*n* = 20), and each group was assigned to a different finishing system. In the first group, the adhesive resin was removed by low-speed TC system (TC group); in the second group, the adhesive resin was removed by low-speed SD system (SD group); third group, the adhesive resin was removed by low-speed diamond system (DB group), (Table 1.). Then, each main group was divided into two subgroups regarding using naked-eye vision or a magnifying loupe for residual cement removal after bracket debonding (naked eye subgroup = NTC; NSD; NDB, magnifying loupe subgroup = MTC; MSD; MDB) (*n* = 10 teeth per group) (Fig. 1).

**Table 1.**
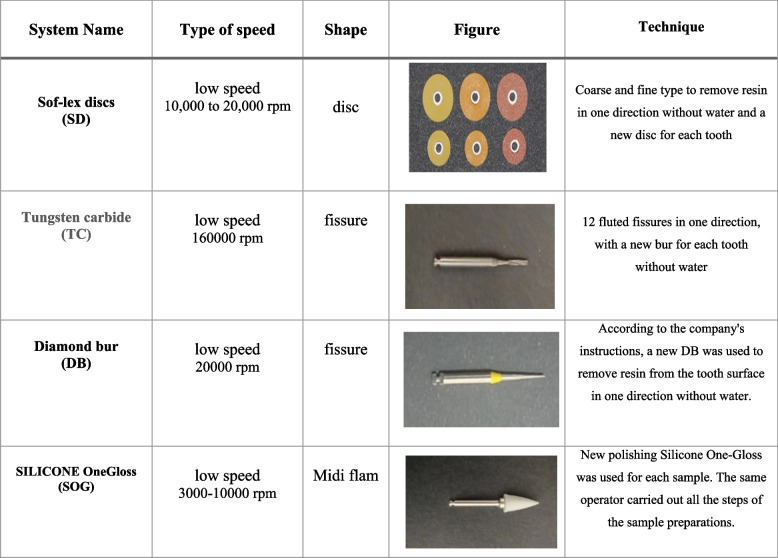
Finishing and polishing system used in the study

### Evaluation of enamel surface roughness (Pre-Bracket Bonding, T0)

All samples were evaluated using a Profilometer (Mitutoyo Surftest SJ-210) at the baseline stage, T0. Three lines were used to measure roughness: the first aligned with the long axis of the crown, and the second and third lines positioned parallel and 0.5 mm mesially and distally to the first line, respectively. Following the manufacturer’s recommendations, each specimen was positioned in the same orientation on the profilometer. A diamond stylus (tip radius: 5 μm) was placed on the tested area and moved across the tooth surface at a static load of 0.4 g and a speed of 0.25 mm/s over a 0.5 mm distance. The average roughness values, expressed in μm, were then recorded [16].

The Scanning Electron Microscope (SEM) was used to examine all samples at baseline T0. The samples were dried using increasing concentrations of ethanol (30%, 50%, 75%, 80%, 90%, 95%, and 100%) and treated with hexamethyldisilazane for 10 min[17]. Then, the specimens were attached to stubs using a resin carbon tape with two sides and coated with gold in a vacuum metalizing machine.

To compare these methods, an assessment of the enamel damage index (EDI) [18] was conducted and the obtained images were evaluated by 3 blinded examiners. The characteristics were then graded by using the EDI developed by Schuler and van Waes [18]. This index includes four scores: **0** Smooth surface without scratches, and perikymata might be visible; **1** Acceptable surface, with fine scattered scratches; **2** Rough surfaces with numerous coarse scratches or slight grooves visible; **3** Surface with coarse scratches, wide grooves, and enamel damage visible to the naked eye.

### Preparation of teeth for bracket bonding and debonding

After cleaning and drying the buccal enamel surfaces of all samples, an acid etching of 37% phosphoric acid gel was applied for 15 s, then rinsed with water spray for 10 s, and air-dried. Then the etched enamel was sealed with the adhesive primer (Transbond XT; 3 M-Unitek, Monrovia, USA), and the light-cured for 5 s. Transbond™ XT adhesive resin was applied to the base of metal brackets; then, the brackets were positioned 4 mm vertically from the tip of the cusp using a straight rod-shaped positioning gauge. The excess adhesive was removed from around the bracket using a dental explorer. Then, the adhesive was light-cured for 40 s on all sides of the bracket with an LED curing unit with a light intensity of 400 mW/cm^2^ according to the manufacturer's recommendations.

One operator carried out the bonding procedure of all brackets, and the samples were stored in distilled water at 37 °C for 24 h before being removed using a debonding plier. The brackets were ultimately debonded by gently squeezing the mesial and distal wings with debonding pliers. The adhesive remnants after bracket debonding were evaluated using the Adhesive Remnant Index (ARI) [19]. The ARI scores range from 1 to 5. A score of 5 indicates that no composite remains on the enamel; 4 indicates less than 10% of composite remains on the tooth surface; 3 indicates more than 10% but less than 90% of the composite remains on the tooth; 2 indicates more than 90% of the composite remains; and 1 indicates all of the composites remains on the tooth.

### Adhesive resin removal and polishing and surface roughness evaluation at T1 and T2

The adhesive remnants were removed using various methods, according to the manufacturer's instructions. In subgroup NTC, low-speed 12-fluted TC burs were used under naked eye vision at a maximum speed of 160,000 rpm. In subgroup NSD, low-speed SD burs were used under naked eye vision at a maximum speed of 20,000 rpm. In subgroup NDB, low-speed DB burs were used under naked eye vision at a maximum speed of 20,000 rpm. In subgroup MTC, low-speed 12-fluted TC burs were used with the aid of an X5 magnifying loupe. Similarly, in subgroups MSD and MDB, low-speed SD and DB burs were used, respectively, with the aid of an X5 magnifying loupe. All burs were applied using light pressure and continuous motion, while the samples were cooled using an air–water syringe. A new bur was used for every two samples to ensure cutting efficiency during adhesive removal and to standardize the procedure. The second surface roughness evaluation (T1) was performed after adhesive removal by the different burs, and the time required for complete resin removal was recorded in seconds.

Final polishing was performed using Silicone OneGloss mounted on a low-speed handpiece at a maximum speed of 10,000 rpm, followed by the third surface roughness evaluation (T2). They were applied using light to moderate pressure for 15–20 s with a constant, continuous, and unidirectional motion to avoid enamel damage, under water cooling, as per the manufacturer's recommendations. A single operator performed all procedures to minimize variability.

### Statistical analysis

The statistical analysis of the data was performed using IBM-SPSS software version 26. The normality distribution of the data was assessed using either the Shapiro–Wilk /Kolmogorov–Smirnov test. A one-way analysis of variance (ANOVA) or Kruskal–Wallis H-test was employed to determine differences between independent groups over time. To determine which means were significantly different from the others, we used the Multiple Comparisons LSD test/ Bonferroni correction.

## Result

The statistical examination of ARI after bracket debonding demonstrates no statistically significant distinctions among the groups, allowing for comparison of all groups for resin removal and enamel polishing. Furthermore, there is no significant difference in the average roughness values among all subgroups at T0 (*p* = 0.994), making all groups comparable (Supplemental Table 1).

The Kruskal–Wallis test demonstrated a statistically significant difference in roughness values between all studied subgroups at both T1 and T2 (*p* < 0.001). After removing residual cement T1, Tukey HSD tests indicated a statistically significant difference between all subgroup pairs, except for NTC-NSD, NTC-MSD, and NSD-MSD, with a *p*-value > 0.05. At polishing stage T2, the Tukey HSD tests showed a statistically significant difference between the NDB subgroup and all other subgroups, as well as between the MDB subgroup and all other subgroups, with a *p* < 0.05 (Table 2,3).

**Table 2:** Enamel surface roughness values at T0, T, and T2 of all groups

Group	TC		SD		DB	
Subgroup	Naked eye	Magnifying loupe	Naked eye	Magnifying loupe	Naked eye	Magnifying loupe
**Roughness at T0**	0.502 ± 0.046	0.501 ± 0.04	0.505 ± 0.036	0.496 ± 0.064	0.509 ± 0.052	0.503 ± 0.039
*P-value	0.994					
**Roughness at T1**	0.936 ± 0.050	0.981 ± 0.026	1.432 ± 0.077	0.849 ± 0.060	0.932 ± 0.052	1.298 ± 0.083
*P-value	0.000					
**Roughness at T2**	0.465 ± 0.067	0.527 ± 0.028	0.501 ± 0.049	0.525 ± 0.039	0.765 ± 0.066	0.603 ± 0.058
*P-value	0.000					

*Abbreviations:* Tungsten carbide (*TC*), Sof-lex discs (*SD*), Diamond burs (*DB*)

Values are mean ± standard deviation. Statistically significant difference at *P* < 0.05 (* Kruskal Wallis test)

**Table 3:** Pairwise test for roughness values at T1 and T2 between subgroups

Contrast	T1	T2
	***P*-values**	***P*-values**
NTC—NSD	0.571	0.672
NTC—NDB	0.000*	0.000*
NTC—MTC	0.025*	0.122
NTC—MSD	1.000	0.140
NTC—MDB	0.000*	0.000*
NSD—NDB	0.000*	0.000*
NSD—MTC	0.000*	0.886
NSD—MSD	0.466	0.910
NSD—MDB	0.000*	0.001*
NDB—MTC	0.000*	0.000*
NDB—MSD	0.000*	0.000*
NDB—MDB	0.000*	0.000*
MTC—MSD	0.039*	1.000
MTC—MDB	0.000*	0.026*
MSD—MDB	0.000*	0.022*

Tukey HSD test. *Statistically significant at *P* < 0.05

**Fig. 2:**
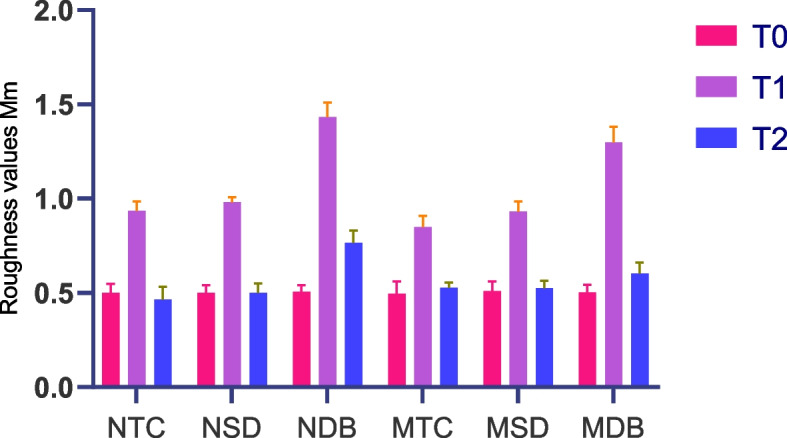
The means roughness values in all subgroups

There was a significant difference in the average roughness values between T0, T1, and T2 in all subgroups (*p* < 0.001, Fig. 2). When comparing the surface roughness, it was found that there was a statistically significant difference in mean roughness values at T1 vs. both T0 and T2 in NDB and MDB (*p* < 0.05.) (Table 4).

**Table 4:** Comparisons of roughness at three time intervals within each subgroup

Variables	Roughness at T0 *P*. value	Roughness at T1 *P*. value	Roughness at T2 *P*. value
NTC	0.000	0.469	0.000
NSD	0.000	1.000	0.000
NDB	0.000	0.000	0.000
MTC	0.000	0.632	0.000
MSD	0.000	1.000	0.000
MDB	0.000	0.004	0.000

When comparing the EDI at T1 and T2, the Kruskal–Wallis H-test showed statistically significant differences in all subgroups. The pairwise comparisons revealed that EDI scores showed a statistically significant difference at T1 and T2 between DB vs. TC and SD (*p* = 0.015) but not between TC vs. SD (*p* = 1.000, Table 5).

**Table 5:** Kruskal–Wallis H-test of EDI in three groups

**Variables**	**TC**		**SD**		**DB**		**KW-H H **[2]	***p*-value**
	**N**	**M**	**N**	**M**	**N**	**M**		
T1	2 (2–2)	2 (2–2)	2 (2–2)	2 (2–2)	3 (3–3)	3 (3–3)	8	0.017
T2	1 (1–1)	1 (1–1)	2 (2–2)	1 (1–1)	3 (3–3)	3 (3–3)	8	0.019
**Pairwise comparisons at T1 and T2**								
TC vs. SD								1.000
TC vs. DB								0.016
SD vs. DB								0.015

**Fig. 3:**
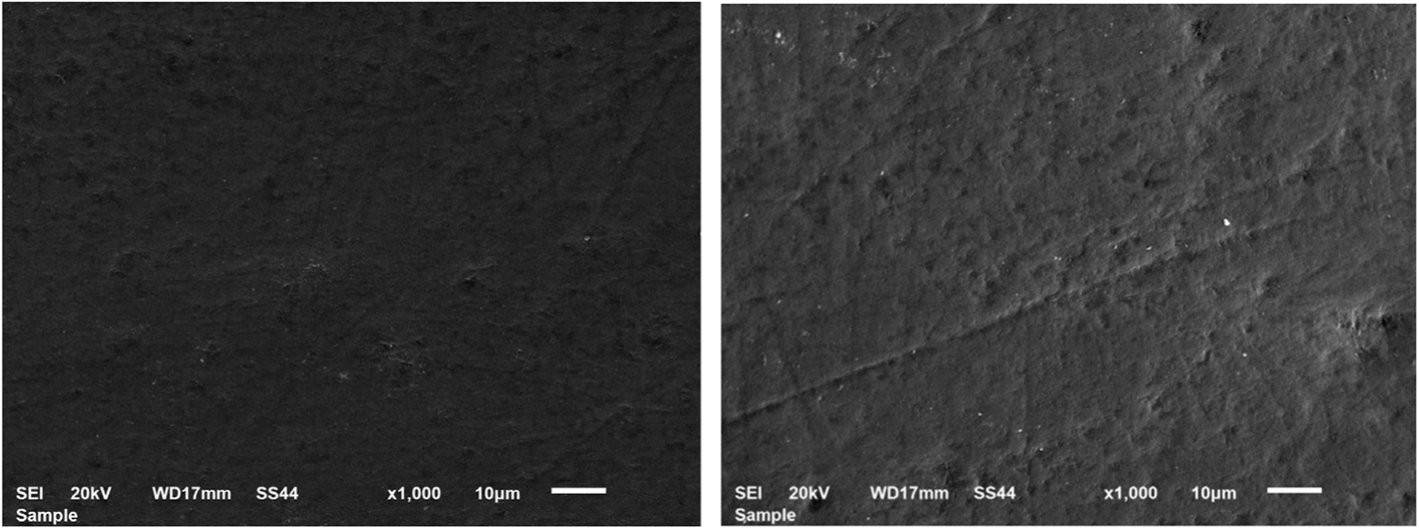
SEM photomicrographs of enamel surface at 1000X magnification before bracket bonding

**Fig. 4:**
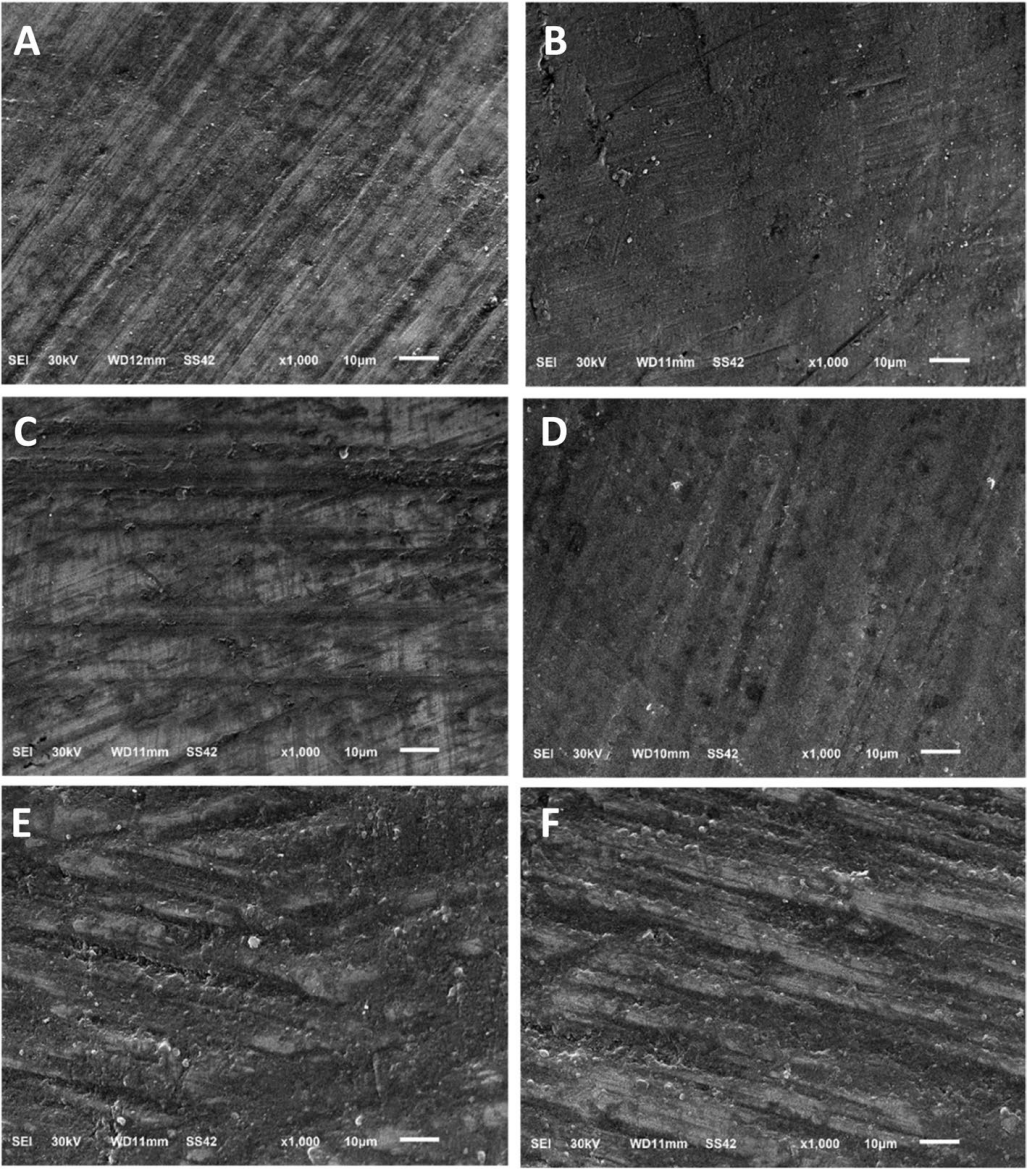
SEM photomicrographs of enamel surface at 1000X magnification after adhesive resin removal: **A**: NTC; **B**: MTC; **C**: NSD; **D**: MSD; **E**: NDB; **F**: MDB

**Fig. 5:**
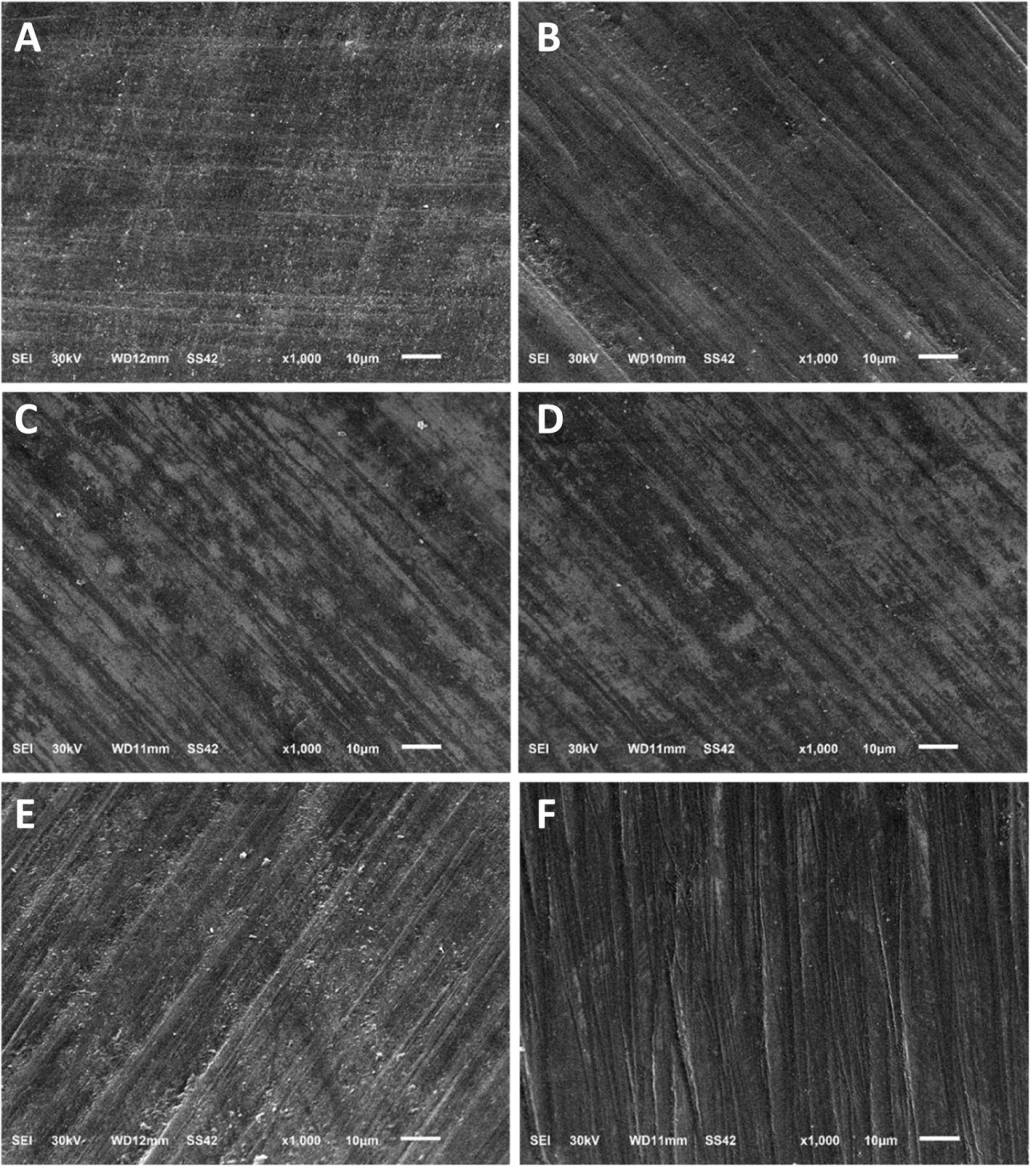
SEM photomicrographs of enamel surface at 1000X magnification after polishing using Silicone One-Gloss: **A**: MTC; **B**: NTC; **C**: MSD; **D**: NSD; **E**: MDB; **F**: NDB

Under SEM, the enamel surface before bonding showed a smooth surface without scratches or grooves (Fig. 3). After adhesive resin removal, the TC and SD groups showed an EDI score 2 in the naked eye group. The EDI score in the magnifying loupe group was 1 in the TC group, and score 2 in SD group. While the DB group showed an EDI score 3 in both the naked eye and magnifying loupe groups (Fig. 4). The sequential polishing using the Silicone One-Gloss produced a smoother and more homogeneous enamel surface in samples treated with TC and SD burs. However, after polishing, the samples treated by DB burs still had a slightly permanent roughened surface with fewer scratches and shallow grooves (Fig. 5).

**Fig. 6:**
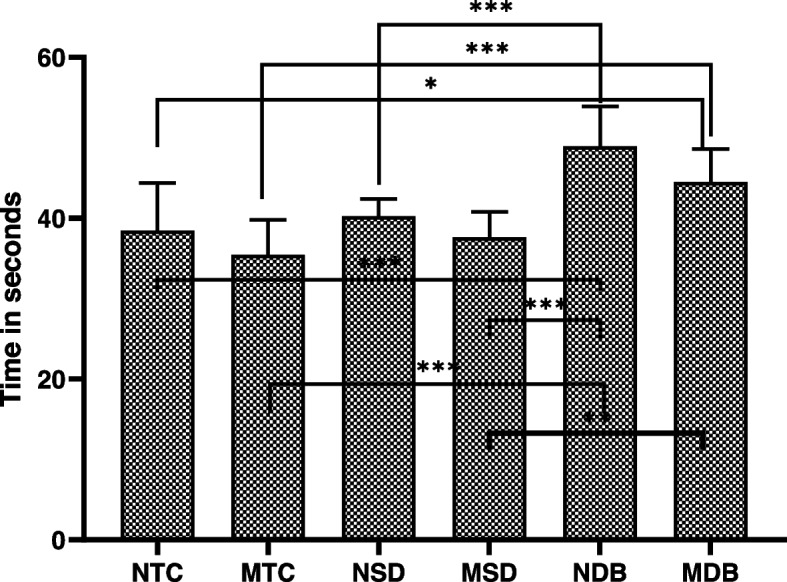
Time consumed for adhesive resin removal in 6 subgroups. This graph applies a *p*-values of multiple comparison procedure to determine which means are significantly different from which others. The method currently being used to discriminate among the means is Fisher's least significant difference (Tukey HSD) procedure. * = *P* < 0.05, ** = *P* < 0.01, *** = *P* < 0.001, ns = *P* > 0.05

A one-way ANOVA test was applied to compare the time consumed for adhesive resin removal for all groups, revealing a statistically significant difference in all subgroups (*p* < 0.001); the TC group took the least amount of working time to remove residual cement (*p* = 0.000). Furthermore, the duration taken for adhesive resin removal was significantly reduced in the magnifying loupe groups compared to the naked eye groups (*p* = 0.001), as shown in Fig. 6.

## Discussion

In orthodontics, the techniques used for attaching and removing orthodontic brackets are critical. Numerous factors impact these procedures, including the choice of adhesive, the bracket debonding instruments, and the methods employed for finishing and polishing to remove adhesive resin [20].

Advances composite resin and adhesive systems have improved the bond between enamel and resin. Nonetheless, this stronger adhesion complicates the removal of residual resin following debonding. The method for removing residual resin is crucial to avoid damage to the enamel surface. Potential damages include cracking, roughness, wear, overheating of the teeth, damage to the pulp, and altered tooth color. Proper removal techniques are vital to maintaining a good aesthetic appearance and surface brightness [21–27]. No technique has been proven to thoroughly and efficiently remove residual adhesives without causing at least minor damage to the enamel [21]. The ideal finishing procedure after debonding should minimize enamel tissue removal and smooth the surface [23]. However, no standard protocol, systematic review, or meta-analysis is available to guide dental practitioners in their daily work routine. Some practitioners use the DB system to reduce chair time, but even with experience and careful use of rotary instruments, enamel crystals can be damaged during mechanical removal.

This study examined the effects of various adhesive removal systems (TC, SD, and DB) on enamel surface roughness after de-bracketing, with and without using a magnifying loupe. Profilometric analysis and SEM were employed to compare the outcomes and measure the time required for adhesive removal. Our findings indicated that the TC group at T1 produced the least enamel surface roughness, followed by the SD group, while the DB group resulted in the highest surface roughness. SEM analysis corroborated these findings, showing an EDI score 2 for TC system and a score 3 for DB in both the naked eye and magnifying loupe groups. These results were consistent with a study conducted by Janiszewska-Olszowska et al.[21] and Sugsompian et al.[24] who found TC system to be more effective in adhesive removal with minimal surface roughness compared to white stones, advising against using white stones due to severe irreversible enamel damage. On the other hand, these findings disagreed with the findings of Mohebi et al.[25] who reported no significant differences in surface roughness between white stones and TC system, possibly due to their use of white stone burs on low-speed handpieces.

Many studies have used different types of burs for polishing procedures. In our experiment, we used Silicone OneGloss bur, which was not found in any previous research that used this bur. Our research findings indicated that the enamel surface in both the TC and SD groups was nearly returned to its pre-treatment condition following the polishing. The results gave a smooth surface and less time than other burs like SD spiral, which needs to change the spiral and consumes time [26–28]. However, the surface roughness decreased significantly in the DB group, but the surface still had a slightly rough surface with shallow grooves. These findings were confirmed by SEM analysis, where the NTC, MTC, and MSD subgroups scored 1 on EDI, while the MDB and NDB subgroups scored 3 on EDI. This finding was in line with studies conducted by Degrazia et al.[29], Janiszewska-Olszowska et al.[21] and Schiefelbein and Rowland [30]. On the other hand, Howell and Weekes[31] disagreed with these results, concluding that medium and fine SD produced a rough surface during polishing. A potential explanation for this discrepancy might be that the SD system was used in a dry condition.

To minimize the risk of enamel damage, we used a low-speed handpiece for adhesive resin removal, which may take longer compared to using a high-speed handpiece [3, 32] The time required to remove adhesive resin with various systems was significantly shorter in the magnifying loupe group than in the naked eye group. The TC system proved to be the least time-consuming for the whole duration of the residual cement removal procedure, followed by SD system. In contrast, the duration of the removal procedure of the remaining cement was the longest with the diamond system. Similar to our results, Tenório et al.[33] Shafiee et al.[34], and Ulusoy [23] showed that the time spent for resin removal with the TC bur was shorter than polymer bur and white stones, respectively. On the other hand, Thawaba et al. [15] reported that. Zirconia burs were more effective for adhesive resin removal than TC systems, causing the least surface roughness and enamel damage.

Regardless of the system chosen for resin removal and finishing, using a magnifying loupe effectively aided in removing adhesive resin with less surface roughness and enamel damage, and it also decreased the time required to remove the adhesive resin. This outcome is in consistent with Baumann et al. [14]. On the other hand, Mohebi et al. [25] found no statistically significant differences in enamel surface roughness and time consumption between the naked eye and loupe magnification groups.

Some limitations of this study have to be mentioned. Firstly, it was carried out in vitro, which does not entirely simulate the conditions inside the mouth. Secondly, we did not evaluate the biological impact of the methods tested on the pulp or dentine. Future studies might benefit from using atomic force microscopy and confocal laser microscopy for a three-dimensional assessment of enamel surface roughness. Lastly, to substantiate our findings and their applicability in clinical practice, further in vivo studies are needed.

## Conclusion

All three burs were clinically effective for removing residual orthodontic adhesive. TC system represents the best one to remove the adhesive resin with a short time consumption comparable to SD burs and can be considered an alternative to DB, which causes severe enamel damage. Regardless of the type of bur used for resin removal, the polishing step by Silicone OneGloss created a smoother surface for the naked eye and was better with a magnifying loupe.

### Supplementary Information

**Supplementary Materials 1.**

## Data Availability

All data generated or analyzed during this study are included in this published article.

## Abbreviations

Μm: Micrometer
ARI: Adhesive Remnant Index
EDI: Enamel Damage Index
SEM: Scanning Electron Microscopy
SOG: Silicone OneGloss
TC: Tungsten carbide bur
SD: Soflex disc
DB: Diamond burs
T0: Time before bracket bonding
T1: Time after adhesive resin removal
T2: Time after polishing

## References

[1] Beniash E, Stifler CA, Sun C-Y, Jung GS, Qin Z, Buehler MJ (2019). The hidden structure of human enamel. Nat Commun.

[2] Arhun N, Arman A. Effects of orthodontic mechanics on tooth enamel: a review. In: Seminars in Orthodontics. Elsevier; 2007. p. 281–91.

[3] Hong YH, Lew KKK (1995). Quantitative and qualitative assessment of enamel surface following five composite removal methods after bracket debonding. Eur J Orthod.

[4] Ryf S, Flury S, Palaniappan S, Lussi A, Van Meerbeek B, Zimmerli B (2012). Enamel loss and adhesive remnants following bracket removal and various clean-up procedures in vitro. The European Journal of Orthodontics.

[5] Iijima M, Yasuda Y, Muguruma T, Mizoguchi I (2010). Effects of CO2 laser debonding of a ceramic bracket on the mechanical properties of enamel. Angle Orthod.

[6] Obata A (1995). Effectiveness of CO~ 2 laser irradiation on ceramic bracket debonding. JOURNAL-JAPAN ORTHODONTIC SOCIETY.

[7] Strobl K, Bahns TL, Wiliham L, Bishara SE, Stwalley WC (1992). Laser-aided debonding of orthodontic ceramic brackets. Am J Orthod Dentofac Orthop.

[8] LEE HW. Effect of various residual adhesive removal methods on enamel surface after bracket debonding: a Systematic Review. 2018.

[9] Tonetto MR, Frizzera F, Porto TS, Jordão KCF, de Andrade MF, dos Santos RSS (2014). Methods for removal of resin remaining after debonding of orthodontic brackets: A literature review. Journal of dental research and review.

[10] Eliades T, Gioka C, Eliades G, Makou M (2004). Enamel surface roughness following debonding using two resin grinding methods. The European journal of orthodontics.

[11] Özer T, Başaran G, Kama JD (2010). Surface roughness of the restored enamel after orthodontic treatment. Am J Orthod Dentofac Orthop.

[12] Christensen GJ (2003). Magnification in dentistry: Useful tool or another gimmick?. J Am Dent Assoc.

[13] Forgie AH, Pine CM, Pitts NB (2001). Restoration removal with and without the aid of magnification. J Oral Rehabil.

[14] Baumann DF, Brauchli L, Van Waes H (2011). The influence of dental loupes on the quality of adhesive removal in orthodontic debonding. Journal of Orofacial Orthopedics/Fortschritte der Kieferorthopädie.

[15] Thawaba AA, Albelasy NF, Elsherbini AM, Hafez AM (2023). Evaluation of enamel roughness after orthodontic debonding and clean-up procedures using zirconia, tungsten carbide, and white stone burs: an in vitro study. BMC Oral Health.

[16] Bhushan B. Surface roughness analysis and measurement techniques. In: Modern tribology handbook, two volume set. CRC press; 2000. p. 79–150.

[17] Thawaba AA, Albelasy NF, Elsherbini AM, Hafez AM. Comparison of Enamel Surface Roughness after Bracket Debonding and Adhesive Resin Removal Using Different Burs with and without the Aid of a Magnifying Loupe. methods. 2022;9:11. 10.5005/jp-journals-10024-3432 37073931

[18] Schuler FS, van Waes H (2003). SEM-evaluation of enamel surfaces after removal of fixed orthodontic appliances. Am J Dent.

[19] Bishara SE, Ortho D, Truiove TS (1990). Comparisons of different debonding techniques for ceramic brackets: an in vitro study: Part I. Background and methods. American Journal of Orthodontics and Dentofacial Orthopedics.

[20] Zarrinnia K, Eid NM, Kehoe MJ (1995). The effect of different debonding techniques on the enamel surface: an in vitro qualitative study. Am J Orthod Dentofac Orthop.

[21] Janiszewska-Olszowska J, Szatkiewicz T, Tomkowski R, Tandecka K, Grocholewicz K (2014). Effect of orthodontic debonding and adhesive removal on the enamel–current knowledge and future perspectives–a systematic review. Med Sci Monit.

[22] Trakyalı G, Özdemir FI, Arun T (2009). Enamel colour changes at debonding and after finishing procedures using five different adhesives. The European Journal of Orthodontics.

[23] Ulusoy Ç (2009). Comparison of finishing and polishing systems for residual resin removal after debonding. J Appl Oral Sci.

[24] Sugsompian K, Tansalarak R, Piyapattamin T (2020). Comparison of the Enamel Surface Roughness from Different Polishing Methods: Scanning Electron Microscopy and Atomic Force Microscopy Investigation. Eur J Dent.

[25] Mohebi S, Shafiee HA, Ameli N (2017). Evaluation of enamel surface roughness after orthodontic bracket debonding with atomic force microscopy. Am J Orthod Dentofacial Orthop.

[26] Gibas-Stanek M, Pihut M (2021). Safe debonding of fixed appliances: a comparison of traditional techniques and lodi devices on different bracket types in terms of enamel cracks, site of bond failure, and bracket reusability. Int J Environ Res Public Health.

[27] Paolone G, Mandurino M, Baldani S, Paolone MG, Goracci C, Scolavino S (2023). Quantitative Volumetric Enamel Loss after Orthodontic Debracketing/Debonding and Clean-Up Procedures: A Systematic Review. Appl Sci.

[28] Tepedino M, Iancu Potrubacz M, Arrizza L, Russo M, Cavarra F, Cordaro M (2020). In vitro shear bond strength of orthodontic brackets after enamel conditioning with acid etching and hydroabrasion. Dent J (Basel).

[29] Degrazia FW, Genari B, Ferrazzo VA, Dos S-P, Grehs RA (2018). Enamel roughness changes after removal of orthodontic adhesive. Dent J (Basel).

[30] Schiefelbein C, Rowland K (2011). A comparative analysis of adhesive resin removal methods. Int J Orthod Milwaukee.

[31] Howell S, Weekes WT (1990). An electron microscopic evaluation of the enamel surface subsequent to various debonding procedures. Aust Dent J.

[32] Panayi NC, Tsolakis AI, Athanasiou AE (2020). Digital assessment of direct and virtual indirect bonding of orthodontic brackets: A clinical prospective cross-sectional comparative investigation. Int Orthod.

[33] Tenório KCS, Feres MF, Tanaka CJ, Augusto MKM, Rodrigues JA, da Silva HDP (2020). In vitro evaluation of enamel surface roughness and morphology after orthodontic debonding: Traditional cleanup systems versus polymer bur. Int Orthod.

[34] Shafiee H-A, Mohebi S, Ameli N, Omidvar R, Akbarzadeh A (2015). Enamel Surface Roughness after Orthodontic Bracket Debonding and Composite Resin Removal by Two Types of Burs. Journal of Dental School Shahid Beheshti University of Medical Science.

