# Supplementary material for: Evaluation of enamel surface integrity after orthodontic bracket debonding: comparison of three different system
Source: BMC Oral Health. 2024 Mar 20;24:358. doi: 10.1186/s12903-024-04138-4 (PMC10953161; doi:10.1186/s12903-024-04138-4)
Supplement: Supplementary file 1 — Supplementary Materials 1. [file 12903_2024_4138_MOESM1_ESM.docx]

**Supplemental Table 1: Comparisons of ARI scores after bracket debonding.**

| Subgroup | Median | Minimum | Maximum | *p* |
| --- | --- | --- | --- | --- |
| NTC | 2 | 1 | 3 | 0.994* |
| MTC | 2 | 1 | 3 |  |
| NSD | 2 | 1 | 4 |  |
| MSD | 2 | 1 | 4 |  |
| NDB | 2 | 1 | 3 |  |
| MDB | 2 | 1 | 3 |  |

*Kruskal-Wallis H-test

Note: 1: All of the composites remains on the tooth, 2: More than 90% of the composite remains, 3 More than 10% but less than 90% of the composite remains on the tooth, 4: Less than 10% of composite remains on the tooth surface, 5: No composite remains on the enamel.
